# Supplementary material for: Genomic landscape of platinum resistant and sensitive testicular cancers
Source: Nat Commun. 2020 May 4;11:2189. doi: 10.1038/s41467-020-15768-x (PMC7198558; doi:10.1038/s41467-020-15768-x)
Supplement: Supplementary file 1 — Supplementary Information [file 41467_2020_15768_MOESM1_ESM.pdf]

Genomic landscape of platinum resistant and sensitive testicular cancers

Loveday et al.

Supplementary Information

Supplementary Tables 1-11
Supplementary Figures 1-7

Supplementary Table 1

Summary clinical data for the ICR2 sample series.

Clinical parameter	Count
Individual/sample counts	
Individuals	26
Samples	40
Age	
Median	32
Range	18-65
Sample type	
Primary (testis)	22
Metastisis	18
Tumour histology (primary)	
Seminoma	5
Nonseminoma	17
Tumour histology (metastasis)	
Seminoma	4
Nonseminoma	14
Sample collected	
Pre-chemotherapy	20
Post-chemotherapy	20
Overall survival	
Alive	17
Deceased	9
Progression after	
1st line platinum CT + resection	7
1st line platinum CT + RT	1
≥2 lines CT + resection	8
≥2 lines CT + resection + RT	10

Abbreviations: CT, chemotherapy; RT, radiotherapy

Supplementary Table 2
Overview of TGCT sample series included in this study.

Centre	Sample series	Platinum response category	Patients	No. primary tumours	No. metastatic tumours	SNV calling platform	CNV calling platform	Sample material type	PMID
ICR	ICR2	Resistant	26	22	18	Exome	MIP	FFPE	NA
	ICR1	Unselected	42	42	0	Exome	Exome	FF	PMID:25609015
DFCI	DFCI	Resistant	22	15	16	Exome	Exome	FFPE	PMID:27905446
		Sensitive	27	27	0				
BROAD	TCGA	Unselected	150	150	0	Exome	Array	FF	PMID:29898407
MSK	MSK	Resistant	89	45	48	Panel (MSK-IMPACT)	na	FFPE + FF	PMID:27646943; PMID:28481359
		Sensitive	65	54	12				
		Unknown	103	66	42				
FDM	FDM	Resistant	107	23	84	Panel (Foundation One)	na	FFPE	PMID:30025711

Abbreviations: MIP, molecular inversion probes; FFPE, formalin fixed paraffin embedded; FF, fresh frozen; na, not applicable.

Supplementary Table 3
Overview of TGCT samples included in this study, by clinical parameters.

Platinum response	Stage	Histology	TCGT DATASETS [664 tumours]					
			Exome [n=290]				Panel [n=374]	
			ICR2 [n=40]	ICR1 [n=42]	TCGA [n=150]	DFCI [n=58]	MSK [n=267]	FDM [n=107]
Platinum Resistant [n=271]	Primary [n=105]	Seminoma [n=34]	5	-	-	3	17	9
		Nonseminoma [n=71]	17	-	-	12	28	14
	Metastasis [n=166]	Seminoma [n=31]	4	-	-	1	12	14
		Nonseminoma [n=134]	14	-	-	14	36	70
		Unclassified [n=1]	-	-	-	1	-	-
Platinum Sensitive/ Unselected [n=285]	Primary [273]	Seminoma [n=117]	-	17	65	15	20	-
		Nonseminoma [n=147]	-	16	85	12	34	-
		Unclassified [n=9]	-	9	-	-	-	-
	Metastasis [n=12]	Seminoma [n=6]	-	-	-	-	6	-
		Nonseminoma [n=6]	-	-	-	-	6	-
Unclassified [n=108]	Primary [n=53]	Seminoma [n=17]	-	-	-	-	17	-
		Nonseminoma [n=49]	-	-	-	-	49	-
	Metastasis [42]	Seminoma [n=4]	-	-	-	-	4	-
		Nonseminoma [n=38]	-	-	-	-	38	-

Supplementary Table 4
Summary of significant small variant logistic regression model variables.

Dependent variable	Independent variable	Estimate	Std. error	z	p	Significance*
KRAS mutation	(Intercept)	-1.20	0.45	-2.64	8.40E-03	**
	Histology: seminoma	1.43	0.30	4.82	1.41E-06	***
	Primary sample site: testis	-1.72	0.48	-3.58	3.39E-04	***
KIT mutation	(Intercept)	-2.67	0.69	-3.84	1.22E-04	***
	Response category: sensitive	1.32	0.39	3.36	7.84E-04	***
	Histology: seminoma	2.77	0.44	6.28	3.33E-10	***
	Primary sample site: testis	-2.23	0.71	-3.14	1.72E-03	**
TP53 mutation	(Intercept)	-0.99	0.87	-1.14	2.54E-01	
	Response category: sensitive	-2.26	1.07	-2.12	3.40E-02	*
	Histology: seminoma	-2.14	1.06	-2.02	4.35E-02	*
	Capture type: panel	1.60	0.79	2.02	4.31E-02	*
	Primary sample site: testis	-2.86	0.54	-5.34	9.42E-08	***
NRAS mutation	(Intercept)	-3.57	0.95	-3.77	1.64E-04	***
	Histology: seminoma	2.43	0.79	3.07	2.17E-03	**
	Primary sample site: testis	-1.87	0.86	-2.18	2.92E-02	*
Putative TGCT driver gene mutation (oncogenes)	(Intercept)	-3.28	0.28	-11.61	<2e-16	***
	Histology: seminoma	0.90	0.38	2.35	0.02	*
RAS pathway mutation	(Intercept)	-0.67	0.42	-1.59	1.12E-01	
	Histology: seminoma	1.54	0.28	5.55	2.89E-08	***
	Primary sample site: testis	-2.08	0.46	-4.58	4.71E-06	***
PI3K/MTOR pathway mutation	(Intercept)	-2.81	0.68	-4.13	< 2e-16	***
	Histology: seminoma	0.88	0.38	2.34	2.49E-02	*
	Capture type: panel	1.18	0.43	2.73	5.32E-03	**

WNT/CTNNB1 pathway mutation	(Intercept)	-2.17	0.26	-8.49	<2e-16	***
	Stage: primary	-1.31	0.61	-2.13	3.33E-02	*
	Response category: sensitive	-2.25	1.11	-2.02	4.34E-02	*

P values are derived from two-sided multivariable logistic regression. All associations are shown with mutation-positive status. Only significant associations are shown. No adjustments were made to account for multiple comparisons.

Supplementary Table 5

Summary of global, arm-level and focal copy number data, by clinical parameters.

Platinum response	Stage	Histology	Ploidy		Fraction aneuploid		Arm-level events		Focal events	
			Median	IQR	Median	IQR	Median	IQR	Median	IQR
Platinum sensitive/unselected [n=134]	Primary [n=134]	Seminoma [n=49]	2.98	2.81-3.23	0.56	0.53-0.62	22	20-24	30	25-33
		Nonseminoma [n=85]	2.75	2.57-2.92	0.51	0.42-0.59	21	18-25	27	23-32
Platinum resistant [n=54]	Primary [n=29]	Seminoma [n=6]	2.81	2.55-2.99	0.59	0.53-0.64	26.5	21.8-30.5	33.5	31.3-37.3
		Nonseminoma [n=23]	2.57	2.47-2.79	0.54	0.46-0.64	20	18-24	30	25-32
	Metastasis [25]	Seminoma [n=5]	2.78	2.50-2.88	0.57	0.51-0.64	23	22-27	31	29-32
		Nonseminoma [n=20]	2.64	2.47-3.93	0.62	0.45-0.70	20.5	16-25	26	19-32.8

Abbreviations: IQR, interquartile range.

Supplementary Table 6

Summary of gene-level copy number event logistic regression model p values.

Gene	Type	Freq.	Histology(seminoma)	Stage(primary)	Platinum_Response(sensitive/unselected)	Sample_Series(ICR1)	Sample_Series(ICR2)	Sample_Series(TCGA)
			P values from logistic regression models					
KRAS	Gain	0.86	0.02	0.62	0.49	0.82	0.66	0.35
RAC1	Gain	0.24	0.01	0.90	0.45	0.87	0.42	0.56
MDM2	Gain	0.21	4.44E-03	1.00	0.81	0.46	0.97	0.91
MYCN	Gain	0.14	0.96	0.25	0.37	0.10	0.02	0.11
PIK3CA	Gain	0.13	0.26	0.55	0.33	0.99	0.04	0.50
KIT	Gain	0.11	0.22	1.00	1.00	1.00	0.22	1.00
FSIP2	Gain	0.08	-	-	-	-	-	-
CTNNB1	Gain	0.05	-	-	-	-	-	-
NRAS	Gain	0.05	-	-	-	-	-	-
Any oncogene (excl. KRAS)	Gain	0.42	-	-	-	-	-	-
KMT2C	Loss	0.01	-	-	-	-	-	-
CREBBP	Loss	0.04	-	-	-	-	-	-
TP53	Loss	0.05	-	-	-	-	-	-
PTEN	Loss	0.02	-	-	-	-	-	-
CBL	Loss	0.20	9.33E-05	0.36	0.99	0.52	0.99	0.94
Any TSG	Loss	0.26	1.40E-05	0.02	0.94	0.31	2.13E-03	0.84

P values are derived from two-sided multivariable logistic regression adjusting for histology, stage, platinum response and dataset. Colours indicate the direction of association, with red indicating a negative association and green indicating a positive association.

Supplementary Table 7

Feature distributions and key associations of copy number signatures in TGCT series TCGA.

CN signatures	Feature distribution TGCT	Feature attributes	Correlation with OC signature	Putative mechanism	Molecular associations & correlations	Orthogonal CN measure associations & correlations
**CN-Sig-1**	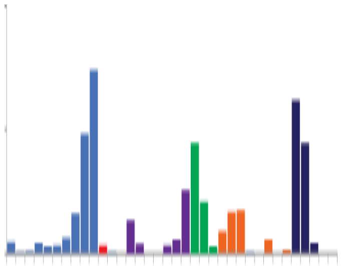	Large segment sizes<br><br>Low number of breakpoints<br><br>0 or 2 breakpoints per chromosome arm	Pearsons correlation, 0.99<br><br>P value, < 2.2e-16	Oncogenic RAS signalling	Higher in KIT and RAS mutated seminoma<br><br>Positively correlated with CBL expression<br><br>Negatively correlated with KRAS expression	
**CN-Sig-3**	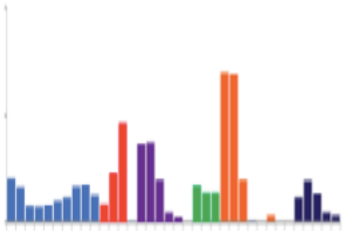	Wide distribution of segment sizes<br><br>Single copy CNVs<br><br>Single copy change point	Pearsons correlation, 0.80<br><br>P value, 5.903e-09	Defective homologous recombination	Lower in KIT and RAS wt seminoma	Negatively correlated with aneuploidy
**CN-Sig-4**	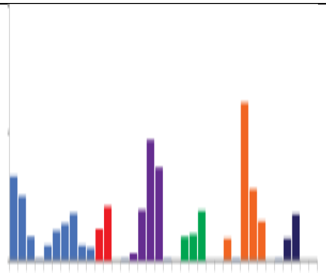	Copy number change points of 2-3<br><br>High copy number states (4-8 copies)	Pearsons correlation, 0.80<br><br>P value, 5.103e-09	Failure of cell cycle control		Positively correlated with aneuploidy

**CN-Sig-5**	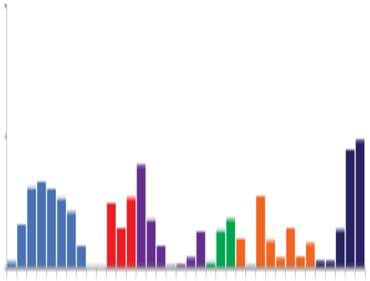	Long chains of oscilating copy number<br><br>Subclonal copy number changes (0.5 copies)<br><br>Large number of breakpoint counts per chromosome arm	Pearsons correlation, 0.87<br><br>P value, 3.299e-12	Chromothriptic-like events through unknown mechanism	Positively correlated with age at diagnosis in seminoma<br><br>Negatively correlated with CBL expression<br><br>Positively correlated with MDM2 expression in nonseminoma	
**CN-Sig-6**	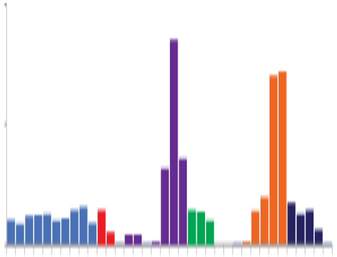	Large copy number change points (6-28)<br><br>High copy number states (8-30 copies)	Pearsons correlation, 0.84<br><br>P value, 9.148e-11	Failure of cell cycle control	Higher in wt seminoma	Positively correlated with aneuploidy

P values are derived from two-sided Pearson's correlation comparing the signature-by-component weight matrices between TGCT series TCGA and OC. No adjustments were made to account for multiple comparisons.

Supplementary Table 8
Copy number signature correlations in TGCT series TCGA.

Variable	Signature	Correlation	P value	Q value	Significance
Age at diagnosis	CN-Sig-1	-0.30	0.06	0.11	
	CN-Sig-3	-0.22	0.18	0.22	
	CN-Sig-4	0.37	0.02	0.05	*
	CN-Sig-5	0.41	0.01	0.05	**
	CN-Sig-6	0.13	0.44	0.44	
KRAS copies	CN-Sig-1	0.15	0.60	0.60	
	CN-Sig-3	0.32	0.27	0.45	
	CN-Sig-4	-0.25	0.39	0.49	
	CN-Sig-5	-0.58	0.03	0.07	*
	CN-Sig-6	0.71	0.00	0.02	**
Chromosome 12p Copies	CN-Sig-1	-0.39	0.01	0.04	*
	CN-Sig-3	-0.24	0.14	0.18	
	CN-Sig-4	0.30	0.06	0.11	
	CN-Sig-5	0.09	0.57	0.57	
	CN-Sig-6	0.51	0.00	0.00	***
Aneuploidy	CN-Sig-1	-0.06	0.53	0.65	
	CN-Sig-3	-0.65	0.00	0.00	****
	CN-Sig-4	0.61	0.00	0.00	****
	CN-Sig-5	-0.04	0.65	0.65	
	CN-Sig-6	0.62	0.00	0.00	****
KIT mRNA expression	CN-Sig-1	0.43	0.01	0.03	**
	CN-Sig-3	0.19	0.24	0.38	
	CN-Sig-4	0.11	0.52	0.52	
	CN-Sig-5	-0.38	0.02	0.05	**
	CN-Sig-6	-0.17	0.31	0.38	
CBL mRNA expression	CN-Sig-1	0.43	0.01	0.03	**
	CN-Sig-3	0.07	0.66	0.66	
	CN-Sig-4	0.14	0.39	0.49	
	CN-Sig-5	-0.32	0.05	0.13	
	CN-Sig-6	-0.19	0.24	0.41	
KRAS mRNA expression	CN-Sig-1	-0.33	0.04	0.10	*
	CN-Sig-3	0.00	0.99	0.99	
	CN-Sig-4	-0.13	0.42	0.53	
	CN-Sig-5	0.15	0.36	0.53	
	CN-Sig-6	0.33	0.04	0.10	*
MDM2 mRNA expression	CN-Sig-1	-0.35	0.00	0.01	**
	CN-Sig-3	-0.07	0.60	0.74	
	CN-Sig-4	0.07	0.56	0.74	
	CN-Sig-5	0.46	0.00	0.00	****
	CN-Sig-6	-0.03	0.81	0.81	

P values are derived from two-sided Pearson's correlation comparing the given feature with signature exposures. Q values represent adjusted P values corrected for multiple comparisons using the Benjamini & Hochberg (BH) method.

Supplementary Table 9

Correlation between copy number signature-by-component matrices in TGCT series.

Series	CN signature 1		CN signature 3		CN signature 4		CN signature 5		CN signature 6	
	**Corr.**	**P value**	**Corr.**	**P value**	**Corr.**	**P value**	**Corr.**	**P value**	**Corr.**	**P value**
ICR1	0.98	2.2E-16	0.75	1.3E-07	0.74	3.3E-07	0.64	2.9E-05	0.84	1.2E-10
ICR2	0.95	2.2E-16	0.33	4.9E-02	0.54	7.1E-04	0.93	3.0E-16	0.47	3.9E-03
DFCI	0.98	2.2E-16	0.49	2.3E-03	0.81	2.1E-09	0.34	3.4E-01	0.46	5.1E-03

Abbreviations: Corr, correlation coefficient. P values are derived from two-sided Pearson's correlation comparing the signature-by-component weight matrices between the indicated datasets and TGCT series TCGA. No adjustments were made to account for multiple comparisons.

Supplementary Table 10

Small variant concordance in patient-matched tumours.

Case ID	Sample 1 ID	Sample 1 stage	Sample 2 ID	Sample 2 stage	Metastasis site(s)	Mutation Counts			% S1 mutations in S2	Jaccard Index
						Shared	S1 only	S2 only		
DFCI_C14	DFCI_C14_T1	Primary	DFCI_C14_T2	Metastasis	RPLN	6	7	13	46%	23.1
DFCI_C21	DFCI_C21_T3	Metastasis	DFCI_C21_T4	Metastasis	Mediastinum; Chest	38	0	12	100%	76.0
DFCI_C21	DFCI_C21_T2	Metastasis	DFCI_C21_T4	Metastasis	Lung;Chest	38	0	11	100%	77.6
DFCI_C21	DFCI_C21_T2	Metastasis	DFCI_C21_T3	Metastasis	Lung; Mediastinum	37	1	2	97%	92.5
DFCI_C21	DFCI_C21_T1	Metastasis	DFCI_C21_T4	Metastasis	Lung; Chest	8	2	5	80%	53.3
DFCI_C21	DFCI_C21_T1	Metastasis	DFCI_C21_T2	Metastasis	Lung; Lung	7	3	2	70%	58.3
DFCI_C21	DFCI_C21_T1	Metastasis	DFCI_C21_T3	Metastasis	Lung; Mediastinum	7	3	2	70%	58.3
DFCI_C3	DFCI_C3_T1	Primary	DFCI_C3_T2	Metastasis	RPLN	1	2	44	33%	2.1
DFCI_C6	DFCI_C6_T2	Metastasis	DFCI_C6_T3	Metastasis	RPLN; Neck	8	1	44	89%	15.1
DFCI_C6	DFCI_C6_T1	Primary	DFCI_C6_T3	Metastasis	Neck	3	9	42	25%	5.6
DFCI_C6	DFCI_C6_T1	Primary	DFCI_C6_T2	Metastasis	RPLN	4	7	3	36%	28.6
DFCI_C7	DFCI_C7_T1	Primary	DFCI_C7_T2	Metastasis	RPLN	4	12	7	25%	17.4
ICR2_C3	ICR2_C3_T2	Metastasis	ICR2_C3_T3	Metastasis	Neck; Mediastinum	52	6	23	90%	64.2
ICR2_C3	ICR2_C3_T1	Primary	ICR2_C3_T3	Metastasis	Mediastinum	14	5	12	74%	45.2
ICR2_C3	ICR2_C3_T1	Primary	ICR2_C3_T2	Metastasis	Neck	18	2	8	90%	64.3
ICR2_C7	ICR2_C7_T1	Primary	ICR2_C7_T2	Metastasis	RPLN	13	6	14	68%	39.4
ICR2_C9	ICR2_C9_T2	Metastasis	ICR2_C9_T3	Metastasis	RPLN; PLN	7	10	8	41%	28.0
ICR2_C9	ICR2_C9_T1	Primary	ICR2_C9_T2	Metastasis	RPLN	5	4	12	56%	23.8
ICR2_C9	ICR2_C9_T1	Primary	ICR2_C9_T3	Metastasis	Pelvic LN	6	5	12	55%	26.1
ICR2_C11	ICR2_C11_T1	Primary	ICR2_C11_T2	Metastasis	RPLN	7	10	0	41%	41.2
ICR2_C12	ICR2_C12_T1	Primary	ICR2_C12_T2	Metastasis	Supraclavicula LN	55	28	86	66%	32.5
ICR2_C13	ICR2_C13_T1	Primary	ICR2_C13_T2	Metastasis	RPLN	14	6	5	70%	56.0
ICR2_C15	ICR2_C15_T1	Primary	ICR2_C15_T2	Metastasis	Cranium	23	18	35	56%	30.3

ICR2_C16	ICR2_C16_T1	Primary	ICR2_C16_T2	Metastasis	Brain	20	5	13	80%	52.6
ICR2_C18	ICR2_C18_T1	Primary	ICR2_C18_T2	Metastasis	RPLN	17	15	101	53%	12.8

Abbreviations: RPLN, retroperitoneal lymph node; PLN, peritoneal lymph node; LN, lymph node.

Supplementary Table 11
ICR2 series tumours used for phylogenetic tree construction.

Overall survival	Case ID	Sample ID	Sample site	Histology	Months since diagnosis	Tree length	Chemotherapy*
Deceased	ICR2_C3	ICR2_C3_T1	Testis	Nonseminoma	0	35	naïve
		ICR2_C3_T2	Neck	Nonseminoma	46	46	BEPx4; TIPx4
		ICR2_C3_T3	Mediastinum	Nonseminoma	63	60	-
Deceased	ICR2_C7	ICR2_C7_T1	Testis	Nonseminoma	0	39	naïve
		ICR2_C7_T2	RPLN	Nonseminoma	12	39	BEPx3
Alive	ICR2_C9	ICR2_C9_T1	Testis	Nonseminoma	0	43	naïve
		ICR2_C9_T2	RPLN	Nonseminoma	36	46	BEPx3
		ICR2_C9_T3	PLN	Nonseminoma	57	42	TIPx2
Alive	ICR2_C11	ICR2_C11_T1	Testis	Seminoma	0	29	naïve
		ICR2_C11_T2	RPLN	Seminoma	12	26	BEPx3
Deceased	ICR2_C13	ICR2_C13_T1	Testis	Nonseminoma	0	42	naïve
		ICR2_C13_T2	RPLN	Nonseminoma	6	40	BEPx3+EPx1
Deceased	ICR2_C15	ICR2_C15_T1	Testis	Nonseminoma	0	38	naïve
		ICR2_C15_T2	Cranium	Nonseminoma	8	90	BEPx3
Alive	ICR2_C16	ICR2_C16_T1	Testis	Nonseminoma	0	38	naïve
		ICR2_C16_T2	Brain	Nonseminoma	19	34	BEPx3+EPx1; TIPx4
Alive	ICR2_C18	ICR2_C18_T1	Testis	Nonseminoma	0	47	naïve
		ICR2_C18_T2	RPLN	Nonseminoma	272	114	BEPx3
Alive	ICR2_C20	ICR2_C20_T1	Testis	Nonseminoma	0	14	BEPx4
		ICR2_C20_T2	RPLN	Nonseminoma	13	71	-

Abbreviations: RPLN, retroperitoneal lymph node; PLN, peritoneal lymph node; BEP, bleomycin, etoposide and platinum; EP, etoposide and platinum; TIP, paclitaxel, ifosfamide and platinum.

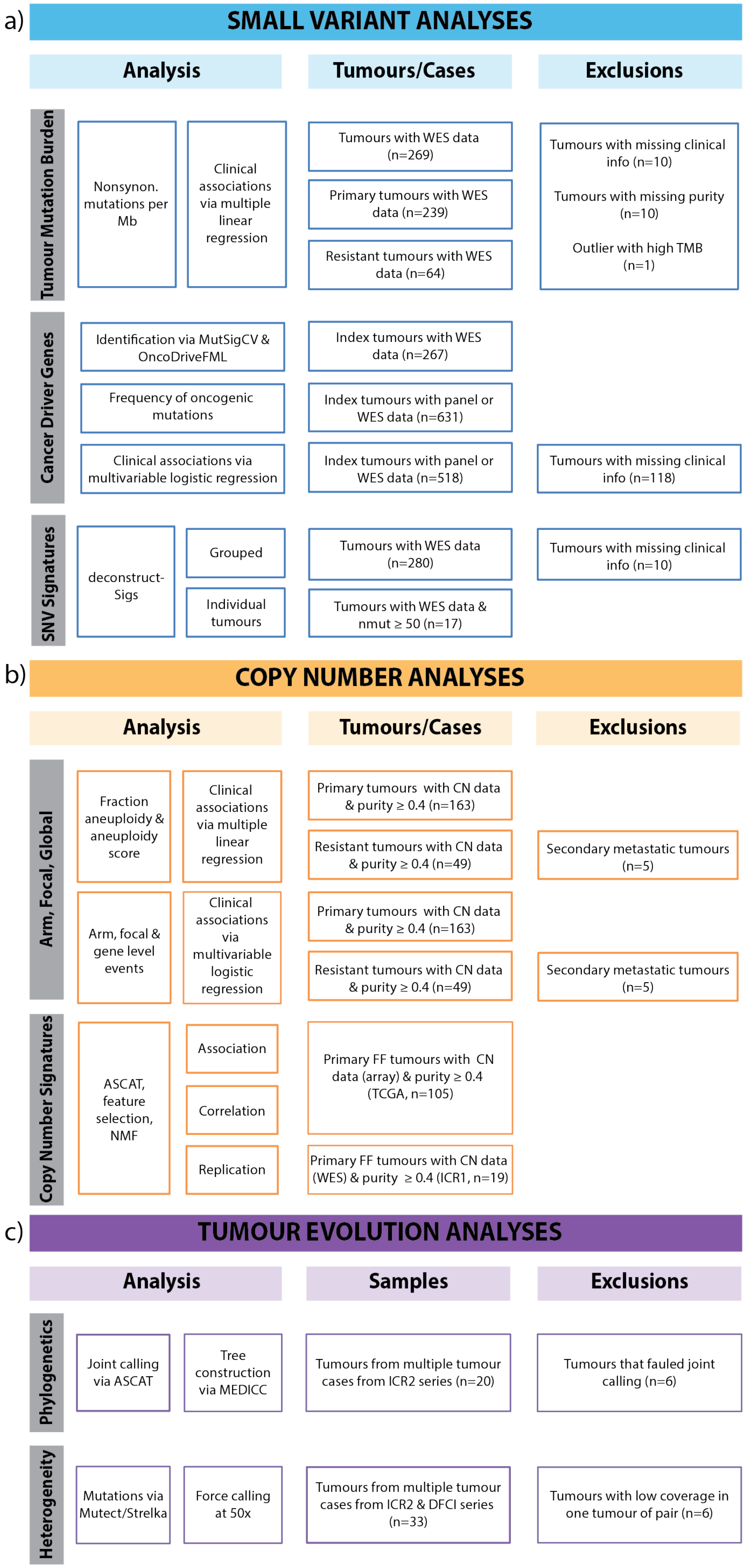
a)
SMALL VARIANT ANALYSES
Analysis
Tumours/Cases
Exclusions
Tumour Mutation Burden
Nonsynon. mutations per Mb
Clinical associations via multiple linear regression
Tumours with WES data (n=269)
Primary tumours with WES data (n=239)
Resistant tumours with WES data (n=64)
Tumours with missing clinical info (n=10)
Tumours with missing purity (n=10)
Outlier with high TMB (n=1)
Cancer Driver Genes
Identification via MutSigCV & OncoDriveFML
Index tumours with WES data (n=267)
Frequency of oncogenic mutations
Index tumours with panel or WES data (n=631)
Clinical associations via multivariable logistic regression
Index tumours with panel or WES data (n=518)
Tumours with missing clinical info (n=118)
SNV Signatures
deconstruct-Sigs
Grouped
Individual tumours
Tumours with WES data (n=280)
Tumours with WES data & nmut ≥ 50 (n=17)
Tumours with missing clinical info (n=10)
b)
COPY NUMBER ANALYSES
Analysis
Tumours/Cases
Exclusions
Arm, Focal, Global
Fraction aneuploidy & aneuploidy score
Clinical associations via multiple linear regression
Primary tumours with CN data & purity ≥ 0.4 (n=163)
Resistant tumours with CN data & purity ≥ 0.4 (n=49)
Secondary metastatic tumours (n=5)
Arm, focal & gene level events
Clinical associations via multivariable logistic regression
Primary tumours with CN data & purity ≥ 0.4 (n=163)
Resistant tumours with CN data & purity ≥ 0.4 (n=49)
Secondary metastatic tumours (n=5)
Copy Number Signatures
ASCAT, feature selection, NMF
Association
Correlation
Replication
Primary FF tumours with CN data (array) & purity ≥ 0.4 (TCGA, n=105)
Primary FF tumours with CN data (WES) & purity ≥ 0.4 (ICR1, n=19)
c)
TUMOUR EVOLUTION ANALYSES
Analysis
Samples
Exclusions
Phylogenetics
Joint calling via ASCAT
Tree construction via MEDICC
Tumours from multiple tumour cases from ICR2 series (n=20)
Tumours that fauled joint calling (n=6)
Heterogeneity
Mutations via Mutect/Strelka
Force calling at 50x
Tumours from multiple tumour cases from ICR2 & DFCI series (n=33)
Tumours with low coverage in one tumour of pair (n=6)

Supplementary Figure 1

Analyses and samples overview. Diagram showing the number of samples in each of the analytic pipelines: a) small variant analyses including TMB, driver gene and SNV signatures; b) copy number analyses including, global measures of aneuploidy (aneuploidy score and fraction aneuploid), arm- and focal-level events, and CN signatures; and c) tumour evolution analyses including phylogenetic tree construction from jointly called CN alterations, and heterogeneity of force-called SNVs. Abbreviations: CN, copy number; WES, whole exome sequencing; TMB, tumour mutation burden.

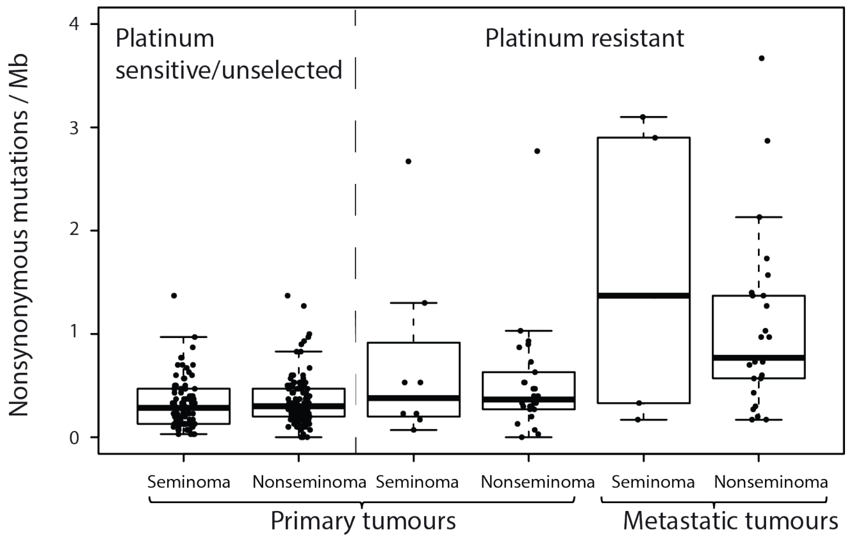

Supplementary Figure 2

Tumour mutation burden in TGCT by histology. Box plot showing mutation burden by histology, subdivided by stage and platinum response. There was no significant difference ($p < 0.05$) between the two histological subtypes in two-sided multiple linear regression when adjusting for sample stage, treatment response, dataset and tumour purity. Boxes show the median ± 25-75th percentiles, whiskers show 1.5 x interquartile range below and above the 25th and 75th percentiles, respectively.

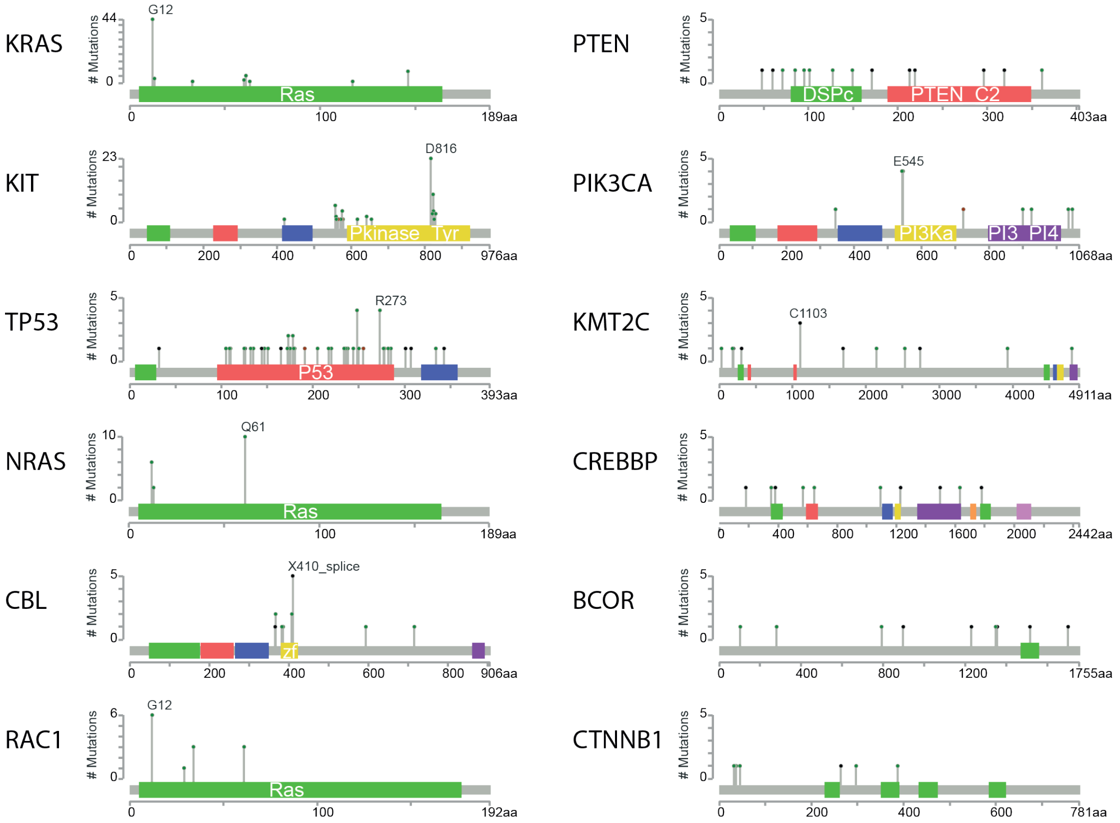

Supplementary Figure 3

Driver gene mutation positions. Schematic representation showing the position of nonsynonymous somatic mutations identified in *KRAS*, *KIT*, *TP53*, *NRAS*, *CBL*, *RAC1*, *PTEN*, *PIK3CA*, *KMT2C*, *CREBBP*, *BCOR*, *CTNNB1*. Green spots, missense/inframe mutations; black spots, nonsense mutations; red spots, frameshift mutations. Coloured blocks indicate the positions of PFAM domains. Key domains indicated: Ras, Ras family domain; P53, P53 DNA binding domain; DSPc, dual specific phosphatase catalytic domain; PTEN_C2, C2 domain of PTEN tumour suppressor gene; PI3Ka, Phosphoinositie 3-kiase family accessory domain; PI3_PI4, Phosphatidylinositol 3- and 4-kinase domain.

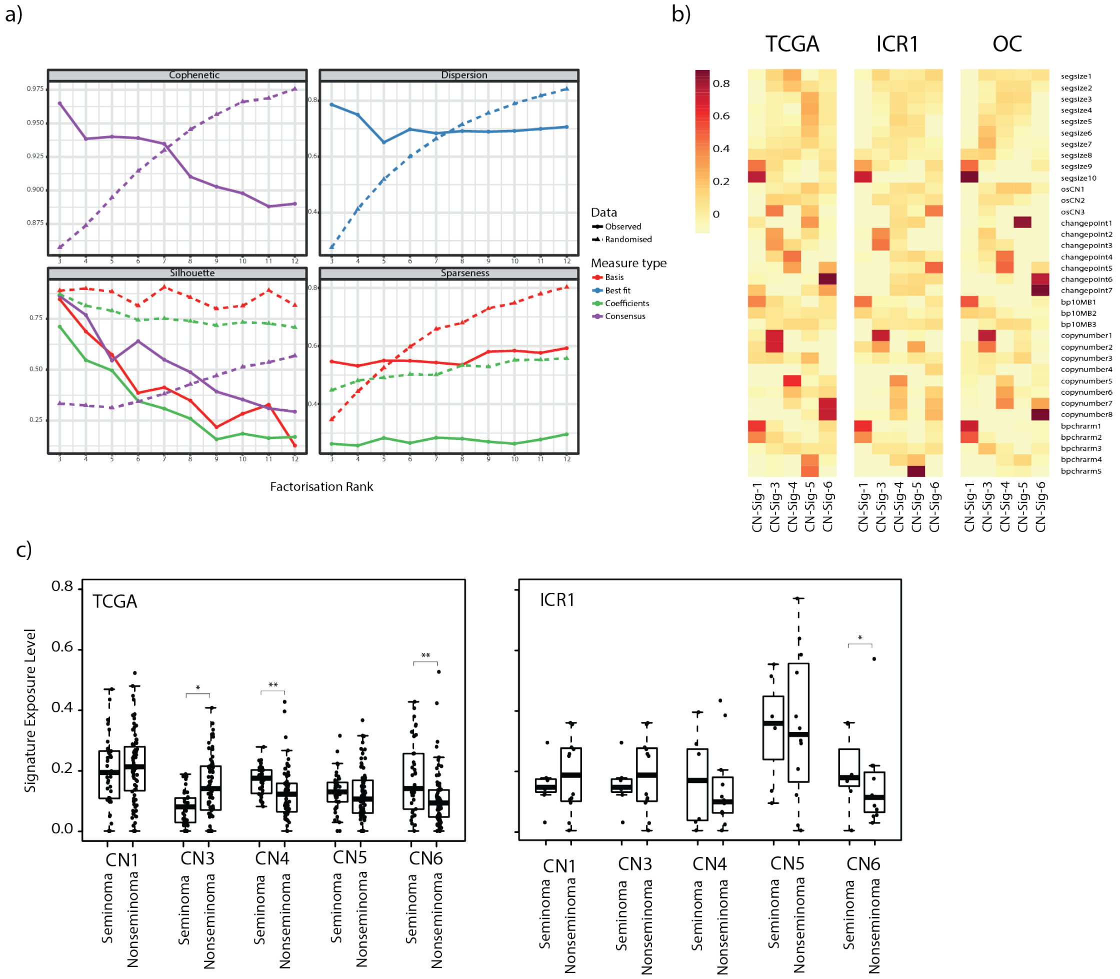

Supplementary Figure 4

Copy Number Signature Identification in TGCT. a) Four measures were used, as previously described, to determine the optimal number of signatures (x-axis) for which to look in the first TGCT series (TCGA, n=105 tumours) (ref. 27). Actual data are represented by solid lines/circles, whereas null results generated by random permutation (n=1000) are represented by dashed lines/triangles. 'Basis' refers to the signature-by-variable matrix. 'Coefficients' refers to the patient-by-signature matrix. 'Consensus' refers to the connectivity matrix of patients clustered by their dominant signature across 1000 runs. Best fit shows the run with the lowest objective score across 1000 runs. The data suggest an optimal signature number of five: higher than this and the sparseness in the signature-by-variable matrix (basis) would be greater than that which could be obtained by randomly shuffling the input matrix. b) Heat map showing the component weights for the five CN signatures identified in TGCT series TCGA (n=105) compared to TGCT series ICR1 (n=19) and ovarian cancer (OC; n=415, as delineated in ref. 27). Associated correlation and p values are shown in Supplementary Tables 7 and 9. c) Box plots showing CN signatures by histology. Boxes show the median ± 25-75th percentiles, whiskers show 1.5 x interquartile range below and above the 25th and 75th percentiles, respectively. Astrisks denote p values derived from two-sided Wilcoxon Rank Sum test: * = $p < 0.05$ and ** = $p < 0.01$.

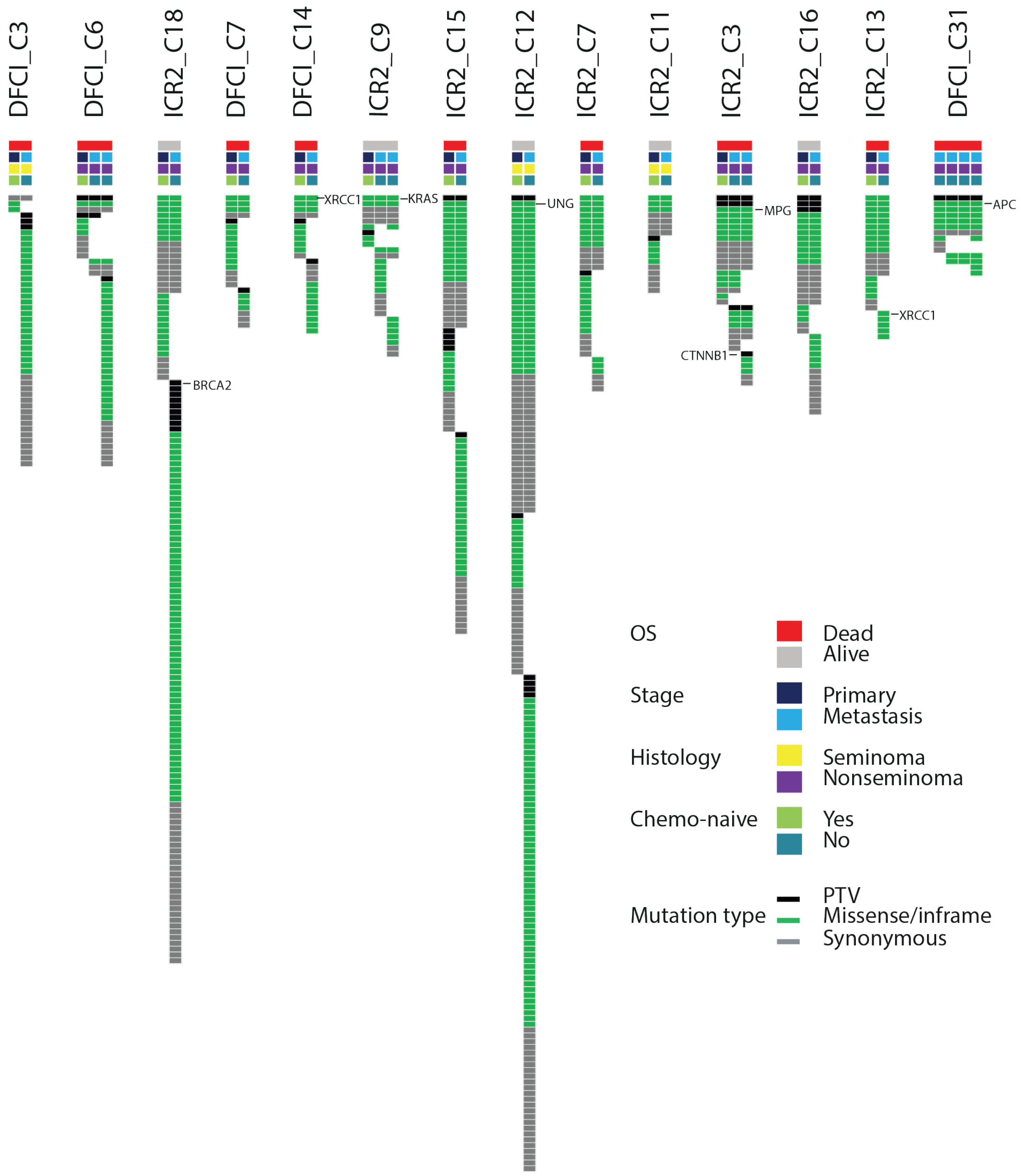

Supplementary Figure 5

Small variant concordance in matched tumours samples. Schematic showing the somatic variants identified in paired primary and metastatic samples (n=33) from 14 TGCT cases from ICR2 and DFCI. Variants identified in a given tumour via the standard pipeline were force-called (see methods) in all other tumours from that individual. Variants are shown if they were a) detected or b) determined absent at 50x. Mutations observed in all tumours from a given individual are termed "truncal". Variants are coloured according to their predicted impact: black blocks indicate nonsense/frameshift (high impact) coding variants; green blocks indicate missense/inframe (moderate impact) coding variants; grey blocks indicate synonymous (low and modifier impact) variants. Mutations highlighted in the schematic comprise those in TGCT driver genes and genes significant in the gene set enrichment analysis.

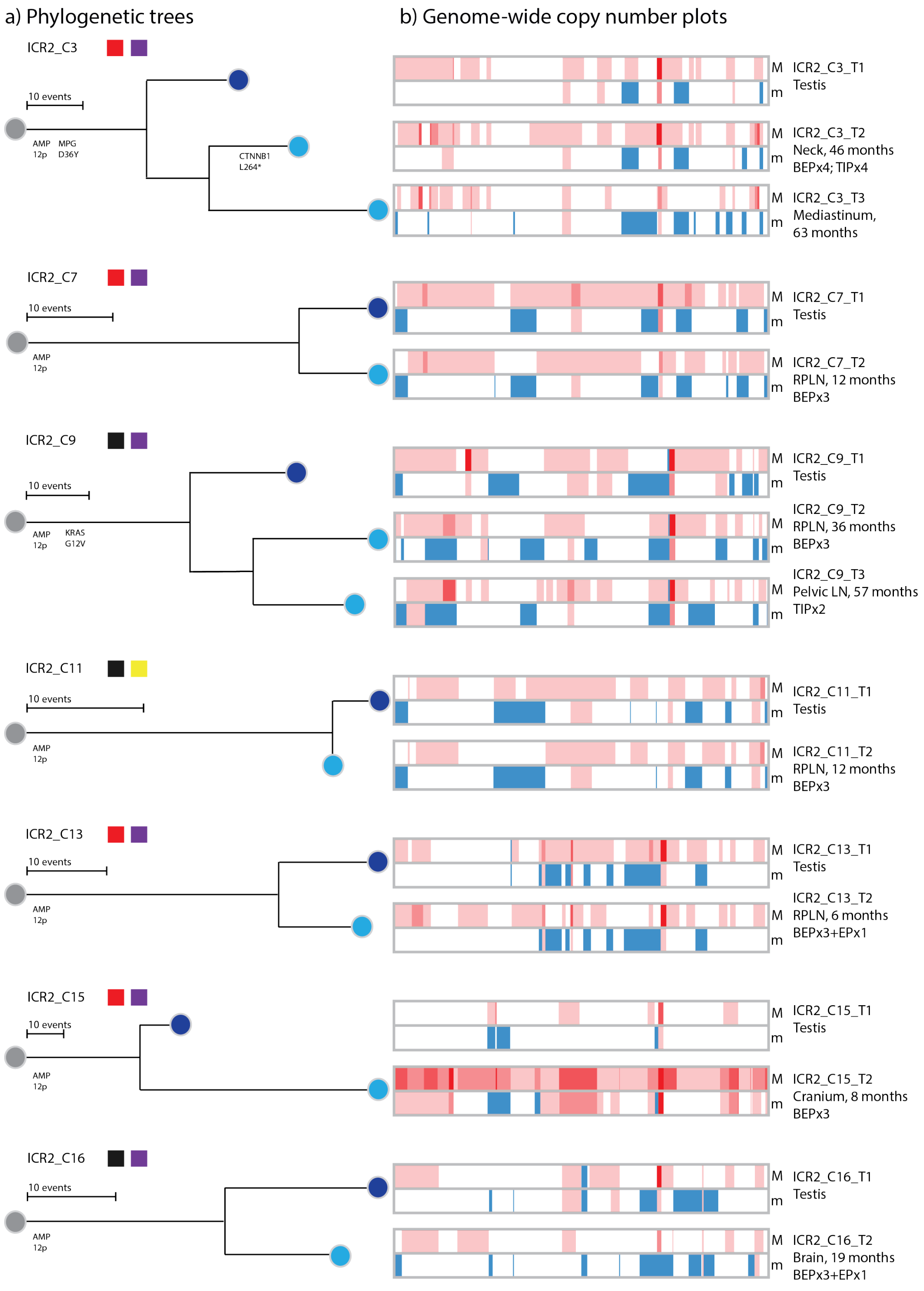
a) Phylogenetic trees
b) Genome-wide copy number plots
ICR2_C3
10 events
AMP 12p
MPG D36Y
CTNNB1 L264*
ICR2_C3_T1
Testis
ICR2_C3_T2
Neck, 46 months
BEPx4; TIPx4
ICR2_C3_T3
Mediastinum, 63 months
ICR2_C7
10 events
AMP 12p
ICR2_C7_T1
Testis
ICR2_C7_T2
RPLN, 12 months
BEPx3
ICR2_C9
10 events
AMP 12p
KRAS G12V
ICR2_C9_T1
Testis
ICR2_C9_T2
RPLN, 36 months
BEPx3
ICR2_C9_T3
Pelvic LN, 57 months
TIPx2
ICR2_C11
10 events
AMP 12p
ICR2_C11_T1
Testis
ICR2_C11_T2
RPLN, 12 months
BEPx3
ICR2_C13
10 events
AMP 12p
ICR2_C13_T1
Testis
ICR2_C13_T2
RPLN, 6 months
BEPx3+EPx1
ICR2_C15
10 events
AMP 12p
ICR2_C15_T1
Testis
ICR2_C15_T2
Cranium, 8 months
BEPx3
ICR2_C16
10 events
AMP 12p
ICR2_C16_T1
Testis
ICR2_C16_T2
Brain, 19 months
BEPx3+EPx1
M
m

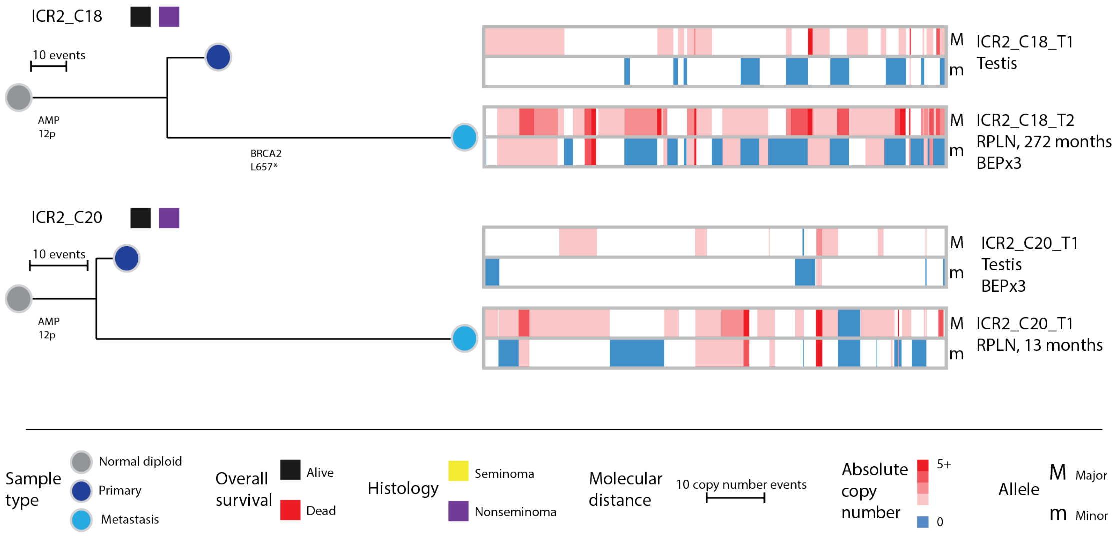

Supplementary Figure 6

ICR2 phylogenetic trees. (A) Phylogenetic trees showing the evolutionary relationship between primary and metastatic tumours. Each tree represents an individual case inferred from allele specific copy number events from a normal diploid sample and two or three tumour samples (total of 20 tumour samples from nine cases from ICR2). Horizontal edges are weighted by the number of somatic copy number aberrations acquired during tumour evolution. Key copy number aberrations are labelled on the appropriate edges, along with manually annotated small variants. Vertical edges are used only to graphically separate nodes and are not weighted. Numbers in brackets represent number of months between tumour and first sample. (B) Copy number profiles used to construct phylogenetic trees. Plots display the absolute copy number counts for the major and minor alleles for each tumour (top and bottom, respectively).

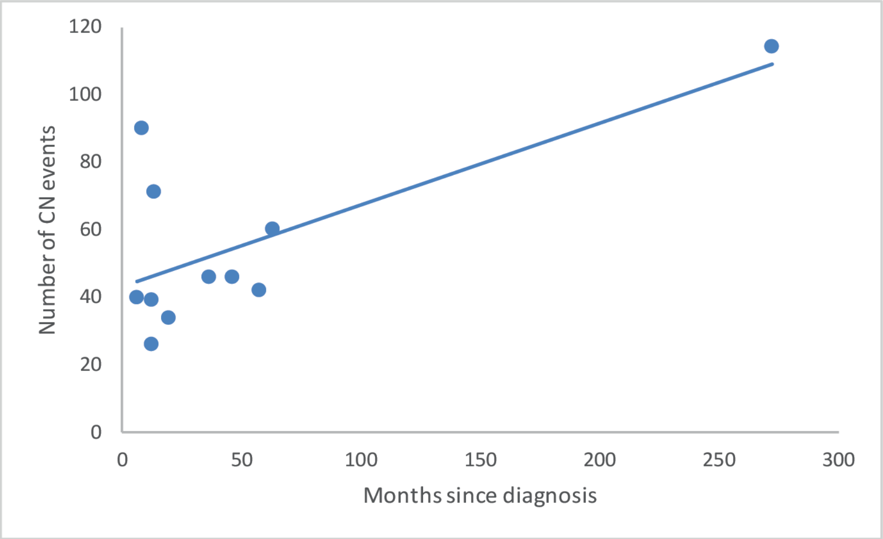

Supplementary Figure 7
CN events vs time since diagnosis. Plot showing the linear relationship between months since diagnosis and number of large-scale copy number events in 11 metastatic tumours from nine cases in the ICR2 series.
