# Supplementary material for: Genomic landscape of platinum resistant and sensitive testicular cancers
Source: Nat Commun. 2020 May 4;11:2189. doi: 10.1038/s41467-020-15768-x (PMC7198558; doi:10.1038/s41467-020-15768-x)
Supplement: Supplementary file 3 — Description of Additional Supplementary Files [file 41467_2020_15768_MOESM3_ESM.pdf]

Description of Additional Supplementary Files

File Name: Supplementary Data 1
Description: This file contains a list of samples included in the study along with relevant clinical parameters and inclusion status for each of the analyses performed.

File Name: Supplementary Data 2
Description: This file contains the exome-wide somatic single nucleotide variants and small indels called in the combined whole exome sequencing series comprising 290 testicular germ cell tumours from 267 cases.

File Name: Supplementary Data 3
Description: This file contains the tumour mutation burden for each testicular germ cell tumour in the combined whole exome sequencing series.

File Name: Supplementary Data 4
Description: This file contains the somatic single nucleotide variants and small indels called in cancer driver genes in the full sample series comprising 664 testicular germ cell tumours from 631 cases.

File Name: Supplementary Data 5
Description: This file contains gene-level summary information of the somatic single nucleotide variants and small indels called in cancer driver genes in the full sample series. The P values presented are derived from two statistical algorithms: MutsigCV, which looks for genes mutated more significantly than expected by chance whilst adjusting for known covariates of mutation rate; and OncodriveFML, which looks for functional impact bias. Q values represent adjusted P values corrected for multiple comparisons using the Benjamini & Hochberg (BH) method.

File Name: Supplementary Data 6
Description: This file contains the estimated clonal status of mutations identified in *KIT*, *KRAS* and *NRAS* in tumours with whole exome sequencing data. We used variant allele fraction, ploidy and purity values to estimate the multiplicity of each variant, that is the number of alleles within a given tumour that carry a particular mutation, using > 0.8 as likely indicative of a mutation being clonal.

File Name: Supplementary Data 7
Description: This file contains the somatic whole arm and focal copy number events identified using GISTIC in the combined whole exome sequencing series where tumour purity was estimated to be greater than or equal to 40% (n = 188 tumours).

File Name: Supplementary Data 8
Description: This file contains a comparison of the frequency of arm level copy number events, comparing a) sensitive vs resistant primary tumours and b) primary and metastatic resistant tumours. P values are derived via two-sided Fisher's exact test. No adjustments were made to account for multiple testing. P values at a nominally statistically significant P < 0.05 are indicated in bold.

File Name: Supplementary Data 9
Description: This file contains a comparison of the frequency of focal copy number events, comparing a) sensitive vs resistant primary tumours and b) primary and metastatic resistant tumours. P values are derived via two-sided Fisher's exact test. No adjustments were made to account for multiple testing. P values at a nominally statistically significant P < 0.05 are indicated in bold.

File Name: Supplementary Data 10
Description: This file contains a summry of the 30 COSMIC SNV signature exposures identified in the combined whole exome sequencing series. Tumours were analysed jointly, grouped at various levels by stage, histology, platinum response and dataset. Tumours were analysed at the individual level only where total SNV count was greater than or equal to 50.

File Name: Supplementary Data 11
Description: This file contains the copy number mutational signature exposures identified in testicular germ cell tumours from the TCGA series with tumour purity greater than or equal to 40% (n=105 tumours).

File Name: Supplementary Data 12
Description: This file contains the results of gene set enrichment analysis performed respectively on truncal and metastatsis-specific nonsynonymous somatic mutations from the matched tumour series (n=33 tumours). Gene set enrichment analysis was performed using the web-based toolset g:Profiler, which uses hypergeometric P values to identify the most significant terms overlapping a queried gene set. Adjustment for multiple comparisons was performed using the g:SCS method.
